# Cemented Sacroiliac Screw Fixation versus Conservative Therapy in Fragility Fractures of the Posterior Pelvic Ring: A Matched-Pair Analysis of a Prospective Observational Study

**DOI:** 10.3390/jcm12185850

**Published:** 2023-09-08

**Authors:** Rene Aigner, Jan Föhr, Julia Lenz, Tom Knauf, Martin Bäumlein, Steffen Ruchholtz, Ludwig Oberkircher, Juliana Hack

**Affiliations:** 1 Center for Orthopaedics and Trauma Surgery, University Hospital Giessen and Marburg GmbH, 35043 Marburg, Germany; 2 Department for Trauma Surgery, Orthopaedic Surgery and Arthroplasty, Medizin Campus Bodensee, 88048 Friedrichshafen, Germany

**Keywords:** geriatric pelvic fracture, sacroiliac screw, conservative therapy, outcome, quality of life

## Abstract

The aim of this study was to compare the outcome of cemented sacroiliac screw fixation to that of conservative treatment in nondisplaced fragility fractures of the sacrum during a 12-month follow-up. Therefore, matched-pair analysis including 40 patients from a previously performed prospective observational study was conducted. Pain was assessed using the visual analogue scale (VAS), functional capabilities and mobility were assessed using the Barthel index, and health-related quality of life (HRQL) was assessed using the EQ-5D questionnaire at 6 weeks, 6 months, and 12 months after the fracture, respectively. No significant differences between the two groups were seen regarding pain. In the operative group, a significantly improved Barthel index was observed after 6 months. A significantly higher HRQL was identified after 6 weeks in the operative group. Their mobility was comparable between the two groups before the fracture; after 6 weeks, mobility was significantly improved in the operative group. After 12 months, no significant differences were found regarding the functional outcome, HRQL or mobility. The 1-year mortality rate was 25% in the conservative group versus 5% in the operative group (not statistically significant). The present study revealed favorable short-term outcomes concerning the functional outcome, HRQL and mobility after sacroiliac screw fixation. After 12 months, the outcomes were similar to those of the patients managed conservatively.

## 1. Introduction

Due to demographic changes, the incidence of fragility fractures of the pelvis (FFP) is increasing [1,2]. An incidence of 22.4 per 10,000 person years in patients aged 60 years or older was calculated in Germany [3]. In contrast to pelvic fractures in younger adults, fragility fractures of the pelvis in the geriatric population mainly occur after low-energy trauma and are associated with the presence of osteoporosis [4].

Stable isolated fractures of the anterior pelvic ring (FFP type I) are usually treated conservatively, while displaced fractures with marked unilateral or bilateral posterior pelvic ring instability (FFP types III and IV) are typically treated operatively [5,6]. Controversy exists, however, regarding the optimal treatment of nondisplaced fractures of the posterior pelvic ring with or without the involvement of the anterior pelvic ring.

Fracture-related pain and immobilization are associated with an increased risk of medical complications [7]. Furthermore, conservative treatment can lead to the development of instability, resulting in fracture progression in up to almost 40% of patients with prolonged pain or limited mobility [8,9].

Recently, other authors have tried to identify the factors associated with therapeutic decisions regarding geriatric pelvic ring fractures [10]. Besides fracture classification, an increased age and poor general health status affect the therapeutic decision, reducing the probability of an operative intervention in the German pelvic injury register [10].

Due to the high prevalence of comorbidities and the increased risk of perioperative complications in geriatric patients, less invasive operative methods such as percutaneous sacroiliac screw fixation are warranted, and recently, have therefore become more relevant in the literature [6,11].

However, only limited information is available regarding the course of the treatment after operative treatment with cemented sacroiliac screw fixation compared to that of conservative therapy. Therefore, the aim of the present study was to compare the outcomes of cemented sacroiliac screw fixation with those of a conservative treatment in a 12-month follow-up.

## 2. Materials and Methods

The present study represents a subgroup analysis of a prospective observational study at a single university trauma center [12]. Consecutive patients with fragility fractures of the pelvis aged 60 years or older were included between 1 June 2012 and 31 December 2016. The exclusion criteria were acetabular fractures, high-energy trauma (ISS ≥ 16) and malignancy-related fractures (e.g., osseous metastases).

The inclusion of patients in the study and data acquisition both began on the day of hospital admission. Further data acquisition was performed daily during hospitalization and at the point of discharge from the hospital. A further clinical examination was performed after 6 weeks. After 6 and 12 months, telephone interviews were performed.

Upon admission, demographic data were collected, including age, gender and ASA classification (American Society of Anesthesiologists) [13]. Functional capabilities were measured using the Barthel index [14], pain level was measured using the visual analogue scale (VAS) and health-related quality of life was measured using the EQ-5D index score with the German value set [15]. The Barthel index before fracture was collected retrospectively upon admission. Mobility was measured using the subclassification of the Barthel index (walking 50 m freely or with crutches (15 points); walking 50 m with a walking frame (10 points), moving only at home with personal help or crutches (5 points) or being immobile (0 points)). Cognitive impairment was assessed using the mini-mental state examination (MMSE) [16].

All patients underwent computed tomography (CT) diagnostics, and fractures were classified according to the FFP classification [5].

The present study represents a subgroup analysis comparing patients treated with isolated sacroiliac screws with matched pairs treated conservatively. All sacroiliac screws in the study population were implanted cement-augmented. A matched-pair analysis with matching according to the fracture type (FFP classification) was performed.

Patients were selected for the matched-pair analysis as follows: All patients from the prospective study who were treated with isolated sacroiliac screw osteosynthesis (*n* = 22) were initially included. These constituted the operative group of the matched-pair analysis. Then, matching partners to each of these 22 patients based on the FFP classification were identified among the conservatively treated patients. For two patients—one patient with an FFP type IIC fracture and one patient with an FFP type IVB fracture—there was no matching partner found in the conservative group, so, finally, 20 surgically and 20 conservatively treated patients were included in matched pair analysis.

The detailed treatment algorithm from our institution has been published before [6]. Basically, conservative treatment with analgesia, physiotherapy and mobilization was performed on all patients with stable fractures, which we assessed all the fractures included in this subgroup analysis. After three to five days, all the patients were reevaluated. The patients who had regained their pre-fracture mobility level at this time and had a pain level <5 on the VAS continued to be treated conservatively. The patients who did not meet these criteria were operated on as soon as possible if the patient’s risk profile permitted surgical therapy and the patient consented to surgery.

All the patients included in this subgroup analysis were allowed to perform pain-adapted full weight bearing at all times, including those treated conservatively from the outset and those treated surgically before and also immediately after surgery.

Statistical analysis was performed using SPSS 22 (Statistical Package for the Social Sciences Version 22, IBM Corp., Armok, NY, USA). For descriptive statistics, the means and standard deviation were determined. Values were tested regarding normal distribution using the Kolmogorov–Smirnov test. Normally distributed values were analyzed using Student’s *t*-test. Otherwise nonparametric tests (Mann–Whitney U test) were used. Dichotomous variables were analyzed using the chi-squared test. Significance was determined at *p* ≤ 0.05.

## 3. Results

A total of 134 patients were included in the prospective observational study. Twenty-two of the patients (16.4%) were treated with isolated cement-augmented sacroiliac screw fixation without further surgical procedures.

**Figure 1:**
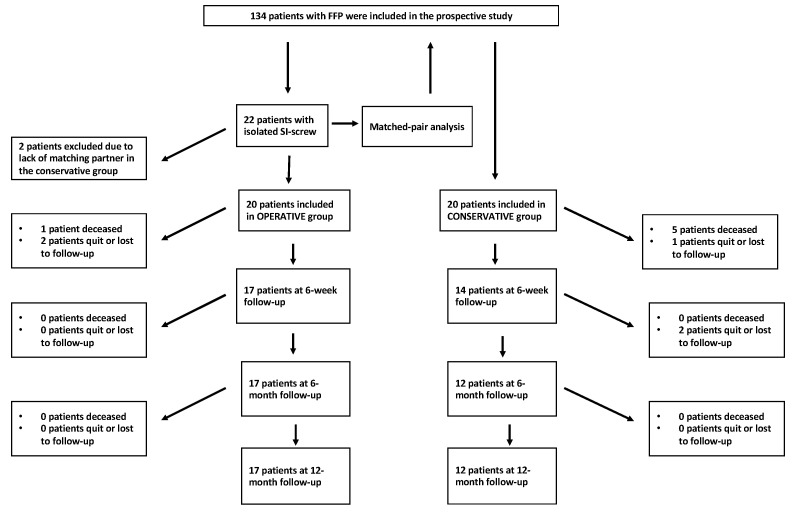
Flowchart of the study.

A matched-pair analysis based on the fracture classification (FFP classification) was performed. Two patients in the operative group had to be excluded due to a lack of a matched partner in the conservative group. Thus, a total of 40 patients were included in the present study. Figure 1 shows the flowchart of the study with the number of patients who underwent different follow-up examinations in both groups.

All fractures were classified as type II fractures, except one in each group, which were slightly dislocated, and therefore, classified as type III. The distribution of the different subtypes of FFP classification is shown in Table 1. There were no significant differences in the demographic parameters between the two groups (Table 2).

While some of the included patients presented directly after the trauma occurred, in others, the trauma had occurred from several days to even weeks before hospital admission. In the conservatively treated group, the trauma occurred on average 12.4 (±27.9) days before admission, which was compared to 12.9 (±16.3) days in the surgically treated group. The difference between both groups was not significant (*p* = 0.08).

In the conservatively treated group, the trauma affected 75% of patients on the day of admission or the day before admission; in the surgically treated group, the trauma affected 40% of the patients on the day of admission or the day before admission.

In the conservative treatment group, one patient died during hospitalization, and four patients died during the 6-week follow-up, while in the surgical treatment group, one patient died between the point of discharge and the 6-week follow-up. Consequently, by the 12-month follow-up, a total of five patients (25%) had died in the conservative group, which was compared to one patient (5%) in the operative group. The difference regarding the 1-year mortality rate was not statistically significant (*p* = 0.182). Furthermore, two patients in the operative group and three in the conservative group were lost during the follow-up, resulting in a follow-up rate (deceased patients excluded) of 87.5% (see Figure 1).

Two patients in each group developed complications during hospitalization. In the operative group, one urinary tract infection and one pulmonary embolism without cardio-respiratory insufficiency occurred. In the conservative group, two patients developed a urinary tract infection, and one patient died during hospitalization due to an acute myocardial infarction.

**Figure 2:**
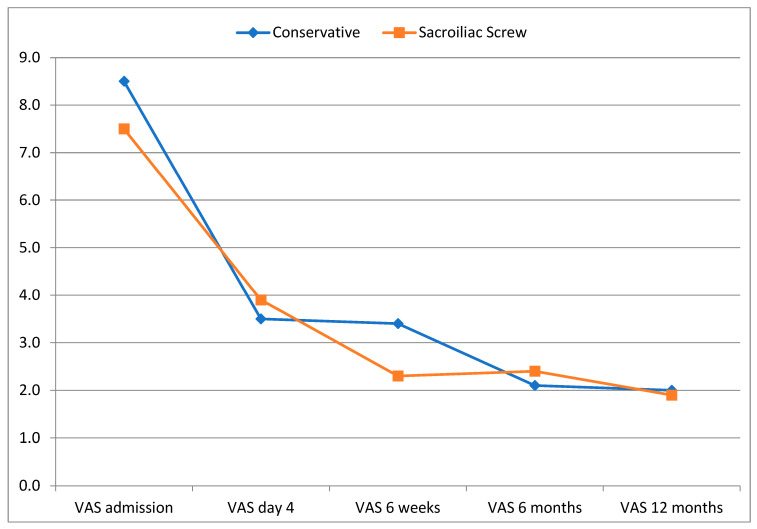
Development of pain (VAS).

Significant pain relief was seen in both groups from the point of admission until day 4 of the hospital treatment (*p* < 0.001). No significant differences between the groups were seen over this time regarding VAS (see Table 3 and Figure 2). However, while patients in the operative group had more pain on day 4 of hospitalization with an average score of 3.9 ± 2.3 vs. 3.5 ± 1.8 in the conservative group (*p* = 0.540), they had an insignificantly lower pain score after 6 weeks, with an average score of 2.3 ± 2.4 vs. 3.4 ± 2.8 in the conservative group (*p* = 0.231).

**Figure 3:**
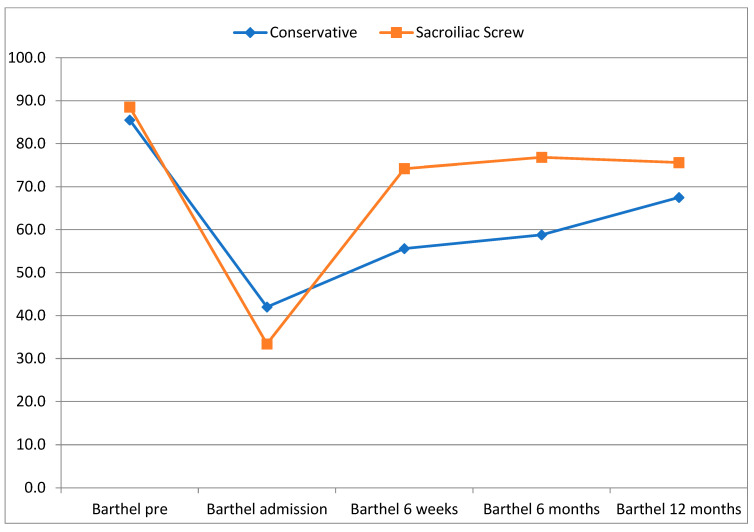
Development of functional status (Barthel index).

Both groups had comparable scores in the Barthel index before the injury. Trends towards an improved Barthel score in the operative group after 6 weeks (74.2 ± 23.5 vs. 55.6 ± 31.2, *p* = 0.051) and a significantly improved Barthel score in the operative group after 6 months (76.8 ± 17.3 vs. 58.8 ± 26.6, *p* = 0.036) were seen. After 12 months, no significant differences were found (see Table 4 and Figure 3).

**Figure 4:**
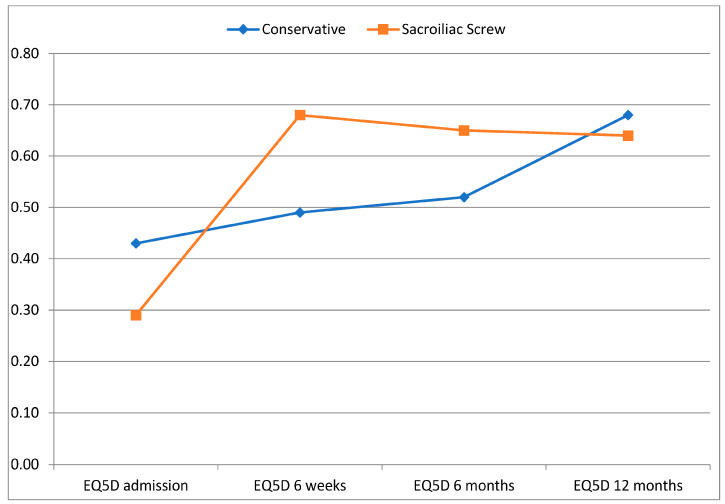
Development of HRQL (EQ-5D).

Upon admission, the patients in the conservative group showed a significantly better HRQL. However, in the 6-week follow-up, a significantly better HRQL was identified in the operative group. No significant differences could be identified after 6 and 12 months (see Table 5 and Figure 4).

**Figure 5:**
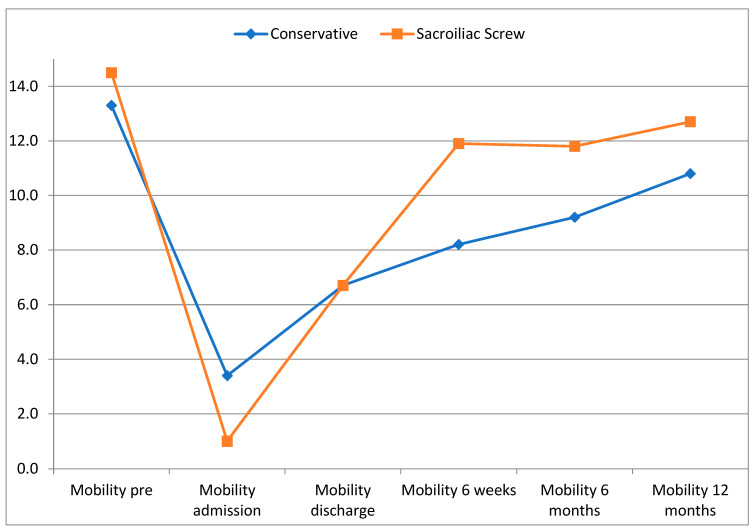
Development of mobility (sub-scale of Barthel index).

Their mobility was comparable between the two groups before the fracture. Upon admission, the patients in the operative group were even more limited in terms of mobility, albeit without statistical significance. On day 4 of hospitalization, significantly fewer patients in the surgical therapy group could be mobilized to stand (*p* = 0.019) and walk (*p* = 0.001). After 6 weeks, however, mobility was significantly improved in the operative treatment group, while no statistically different mobility scores were identified after 6 or 12 months (see Table 6 and Figure 5).

## 4. Discussion

The present study revealed favorable outcomes in terms of the functional outcome, HRQL and mobility after surgical fixation with percutaneous cemented sacroiliac screws in the short term. After 12 months, the outcomes were similar to those of the conservatively managed patients. However, an insignificantly increased 1-year mortality rate was found in the conservative group, which could potentially be a consequence of worse short-term outcomes.

With increasing incidence due to demographic changes, the optimal treatment of pelvic fractures involving the posterior pelvic ring has become more relevant. Several older studies reported acceptable outcomes after conservative management [17,18,19]. However, other authors report poor outcomes after the conservative treatment of unstable posterior pelvic ring fractures, including additional displacement, reduced functional outcome and mobility and increased pain levels [8,9,20,21].

In this study, a short operation time was noted, which is comparable to that in prior research [22]. Furthermore, a significantly shorter length of hospital stay associated with conservative management was noted, which is also in accordance with prior research [23].

In the present study, a significantly reduced pain score was recorded in both treatment groups. The pain scores did not differ between the treatment groups at any point during the follow-up. Höch et al. showed a significantly reduced pain score (VAS) upon discharge after the surgical treatment with cemented sacroiliac screws compared to that upon admission [22]. In their study including 34 patients, the pain had slightly worsened again by the 1-year follow-up. In addition, Hopf et al. showed a significantly reduced pain score after percutaneous sacroiliac screw fixation in elderly patients [24]. In their retrospective study comparing the results of percutaneous sacroiliac screws with those of conservative treatment, Chen et al. report significantly better pain relief 1 and 12 months after operative management [20]. While this pain relief was similar to the results of the present study in the operative therapy group, persistently higher pain levels were noted after conservative management, with persistently worse pain after 12 months [20]. In contrast, in our study, comparable pain relief was also achieved with conservative management. A higher pain level was only seen after 6 weeks, although this was not statistically significant. In line with the previous literature, at the final follow-up, no differences regarding the pain level were identified [23].

Recently, conservative management has been associated with poor functional outcomes in the literature [4,8,9]. Comparing the autonomous state before and after pelvic fractures, a high loss of autonomy was observed in a study by Maier et al. The number of patients needing daily assistance nearly doubled [4]. In line with the results of our study, better functional results were reported after percutaneous sacroiliac screw fixation compared to those of conservative treatment [20]. However, in the present study, differences in the Barthel score equalized by the 1-year follow-up.

Grubor et al. showed the faster mobilization of surgically treated patients compared to that achieved with the conservative treatment [21]. Although comparison with the results of the current study is limited due to different surgical methods, this is in line with our results, as a significant improvement in mobility was shown after 6 weeks. After 6 and 12 months, mobilization within the treatment groups again reached similar levels without significant differences.

While Chen et al. did not find significant differences in the SF 36 physical and mental summary scores, a significant improvement was found in the categories “general health” and “mental health after operative treatment” [20]. The present study showed a significantly improved health-related quality of life among the patients treated with operative fixation after 6 weeks. However, no significant differences were found after 6 or 12 months. In accordance with the results of our study, Höch et al. did not identify differences regarding the HRQL in the long-term follow-up (2 years) [23].

Mortality rates up to 27% after 1 year have been described in geriatric patients suffering from pelvic fractures [25]. After the non-operative management of low-energy pelvic fractures among patients over the age of 70 years, a mortality rate comparable to those of patients suffering from femoral neck fractures was described [26]. The present study showed reduced mortality in patients with operative treatment. Although this difference was not statistically significant, most probably due to the small sample size, it still seems clinically relevant. Since immobilization is a known risk factor for mortality, the increased mortality in the conservative group might be, amongst other factors, caused by reduced mobility and a worse functional status in this group in the short term. The reduced mortality rate after surgical treatment is in line with the literature. Buller et a. showed a decline in mortality, paralleling an increase in the proportion of patients treated with surgical fixation in a population-based study [1]. Höch et al. described a significantly increased survival rate for operatively treated patients. However, comparison to our results is limited as they also included patients given surgical treatments other than isolated sacroiliac screws [23].

In summary, the results of this study suggest that patients seem to have a significant benefit of surgical stabilization in the short term, while the outcomes equalize at the 12-month follow-up. Furthermore, an insignificantly increased mortality rate could be observed in the conservative group, which has to be proven in the future with prospective studies. As all deaths occurred in the first 6 weeks in this study, the short-term outcomes seem to be crucial in the geriatric population.

This study is limited by different factors. First of all, it contains a relatively small sample size. Furthermore, the results of the current study have to be interpreted with caution because no randomization was performed for the allocation of treatments in this prospective observational trial. Matched-pair analysis was performed retrospectively. Although the treatments were performed following a standardized treatment algorithm, the choice of treatment was still made via a subjective decision of the treating physician together with the patient.

Another limitation is the fact that the surgically treated patients were not operated on immediately, but rather, on average, 5 days after the trauma, which may have negatively influenced the results of the surgical group. It is all the more remarkable that mobility in the operative group was significantly better than in it was the conservative group at 6 weeks, despite the potential disadvantage of delayed operative therapy and despite the fact that mobility in the operative group was significantly worse than it was in the conservative group on day 4 of the inpatient stay.

A strength of the current study is that detailed data were collected prospectively over a 12-month follow-up. Furthermore, fracture-type-based matched-pair analysis was performed to identify differences among the treatment groups.

## 5. Conclusions

The results of the present matched-pair analysis show superior outcomes with regard to the HRQL and mobility at 6 weeks and with regard to the functional status at 6 months after the surgical treatment of fragility fractures of the pelvis involving the posterior pelvic ring. However, after 12 months, no significantly different outcomes were found. Nevertheless, an insignificantly increased 1-year mortality rate was found in the conservative group, which could, amongst other factors, be caused by worse short-term outcomes. Future prospective randomized trials are warranted to validate these results.

## Figures and Tables

**Table 1:** Distribution of FFP subtypes.

FFP Classification	Conservative	Sacroiliac Screw
IIa	*n* = 4 (20%)	*n* = 4 (20%)
IIb	*n* = 11 (55%)	*n* = 11 (55%)
IIc	*n* = 4 (20%)	*n* = 4 (20%)
IIIb	*n* = 1 (5%)	*n* = 1 (5%)

**Table 2:** Demographic parameters.

	Conservative	Sacroiliac Screw	*p* Value
Age	82.1 ± 5.5 years	80.8 ± 6.8 years	0.511
Gender (male/female)	2/18	4/16	0.376
BMI	25.6 ± 4.4 kg/m^2^	26.3 ± 4.7 kg/m^2^	0.741
ASA score	2.9 ± 0.9	2.7 ± 0.5	0.531
MMST	22.3 ± 6.4	23.7 ± 5.9	0.533
Barthel index pre-fracture	85.5 ± 17.7	88.5 ± 12.2	0.925
Mobility pre-fracture	13.3 ± 2.9	14.5 ± 1.5	0.277
Hospital stay	8.6 ± 2.9	13.3 ± 4.1	<0.001
Operation time	-	38.4 ± 16.5	
Admission—surgery (d)	-	4.9 ± 3.4	

**Table 3:** VAS.

	Conservative	Sacroiliac Screw	*p* Value
VAS admission	8.5 ± 1.5	7.5 ± 2.5	0.258
VAS day 4	3.5 ± 1.8	3.9 ± 2.3	0.540
VAS 6 weeks	3.4 ± 2.8	2.3 ± 2.4	0.231
VAS 6 months	2.1 ± 2.7	2.4 ± 3.1	0.981
VAS 12 months	2.0 ± 2.7	1.9 ± 2.9	0.905

**Table 4:** Barthel index.

	Conservative	Sacroiliac Screw	*p* Value
Barthel pre-injury	85.5 ± 17.7	88.5 ± 12.2	0.925
Barthel admission	42.0 ± 24.8	33.4 ± 15.9	0.209
Barthel 6 weeks	55.6 ± 31.2	74.2 ± 23.5	0.051
Barthel 6 months	58.8 ± 26.6	76.8 ± 17.3	**0.036**
Barthel 12 months	67.5 ± 24.7	75.6 ± 18.0	0.322

**Table 5:** EQ-5D index.

	Conservative	Sacroiliac Screw	*p* Value
EQ-5D admission	0.43 ± 0.24	0.29 ± 0.15	**0.026**
EQ-5D 6 weeks	0.49 ± 0.25	0.68 ± 0.23	**0.030**
EQ-5D 6 months	0.52 ± 0.25	0.65 ± 0.27	0.204
EQ-5D 12 months	0.68 ± 0.22	0.64 ± 0.30	0.681

**Table 6:** Mobility.

	Conservative	Sacroiliac Screw	*p* Value
Mobility pre-fracture	13.3 ± 2.9	14.5 ± 1.5	0.277
Mobility admission	3.4 ± 5.0	1.0 ± 3.1	0.166
Mobility discharge	6.7 ± 5.6	6.7 ± 4.9	0.986
Mobility 6 weeks	8.2 ± 4.7	11.9 ± 4.2	**0.019**
Mobility 6 months	9.2 ± 4.5	11.8 ± 3.0	0.145
Mobility 12 months	10.8 ± 4.2	12.7 ± 3.1	0.263

## Data Availability

The data presented in this study are available within the article.
